# A Monocular Vision Sensor-Based Obstacle Detection Algorithm for Autonomous Robots

**DOI:** 10.3390/s16030311

**Published:** 2016-03-01

**Authors:** Tae-Jae Lee, Dong-Hoon Yi, Dong-Il “Dan” Cho

**Affiliations:** 1 Department of Electrical and Computer Engineering, Automation and Systems Research Institute (ASRI), Seoul National University, Seoul 151-742, Korea; ltj88@snu.ac.kr (T.-J.L.); ydh01@snu.ac.kr (D.-H.Y.); 2 Inter-University Semiconductor Research Center (ISRC), Seoul National University, Seoul 151-742, Korea

**Keywords:** obstacle detection, monocular vision, segmentation

## Abstract

This paper presents a monocular vision sensor-based obstacle detection algorithm for autonomous robots. Each individual image pixel at the bottom region of interest is labeled as belonging either to an obstacle or the floor. While conventional methods depend on point tracking for geometric cues for obstacle detection, the proposed algorithm uses the inverse perspective mapping (IPM) method. This method is much more advantageous when the camera is not high off the floor, which makes point tracking near the floor difficult. Markov random field-based obstacle segmentation is then performed using the IPM results and a floor appearance model. Next, the shortest distance between the robot and the obstacle is calculated. The algorithm is tested by applying it to 70 datasets, 20 of which include nonobstacle images where considerable changes in floor appearance occur. The obstacle segmentation accuracies and the distance estimation error are quantitatively analyzed. For obstacle datasets, the segmentation precision and the average distance estimation error of the proposed method are 81.4% and 1.6 cm, respectively, whereas those for a conventional method are 57.5% and 9.9 cm, respectively. For nonobstacle datasets, the proposed method gives 0.0% false positive rates, while the conventional method gives 17.6%.

## 1. Introduction

One of the goals in robotics is to develop a mobile robot that can act autonomously in the real world. For this purpose, detecting obstacles in front of the robot that are dangerous or impossible to traverse is a prerequisite for motion planning [1,2,3,4]. Obstacle detection is a particularly important issue for autonomous service robots such as robotic vacuums or monitoring robots, because they must drive through cluttered environments and must be able to traverse every nook and cranny. They often get stuck on obstacles such as a tangled wire or a garment and stop operating altogether. Although innumerable studies have been done on obstacle detection and avoidance, it remains an unresolved problem, especially when considering the real environment and cost.

Most state-of-the-art commercialized autonomous robots such as home service robots rely on contact or range data for obstacle detection. The well-known robotic iRobot Roomba vacuum [5] uses an infrared sensor array and bump sensors for obstacle detection. The Neato Botvac robot [6] uses laser range finders and bump sensors, and the Dyson 360 Eye robot [7] uses an infrared sensor array. The Samsung POWERbot robot [8] uses an infrared sensor array and bump sensors, and the LG Roboking robot [9] uses an ultrasonic sensor array. The DR Robot Sentinel series robot [10], which is a remote monitoring robot, uses an infrared sensor and an ultrasonic sensor array. However, none of these sensors are ideal. The bump sensors can detect obstacles only after physically bumping into them, which may damage furniture or other household items. Infrared sensors suffer from a narrow field of view and nonlinear reflectance characteristics. Ultrasonic sensors suffer from specular reflections and poor angular resolution, which lead to incorrect detection. Laser rangefinders provide better resolution but are power and cost intensive. In addition to their individual shortcomings, all range-based sensors have difficulty in detecting small or flat objects on the ground. Reliable detection of such objects requires high measurement accuracy and precise calibration.

In contrast, vision-based systems provide rich information about the environment and have become promising alternatives, especially considering the current availability of low-cost image sensors and high-performance processors. Of the various vision-based approaches, monocular vision-based approaches are the most suitable for various reasons such as low cost, light weight, and short processing times. This work thus presents a monocular vision-based obstacle detection method. The goal of this research is to detect various types of low obstacles that are difficult to distinguish from the floor. Especially, this work focuses on the situation where the camera is low above the floor. In various robot platforms such as robotic vacuums or small monitoring robots, the camera cannot be installed high above the floor. In this case, extracting cues for distinguishing between the floor and obstacle regions in an image from a conventional point tracking method becomes extremely difficult. The detailed situation when the camera is low above the floor is analyzed in Section 2. In addition, we focus on minimizing the false positives related to nonobstacle images.

In this work, we focus on inverse perspective mapping (IPM) to obtain geometric cues in obstacle detection. For further performance improvement, a vertical plane model is adopted at the IPM stage. At the next step, appearance-based obstacle segmentation using the IPM results and the learned floor appearance model is applied. Next, the shortest distance between the robot and the obstacle to be avoided is calculated. We evaluated the proposed method by applying it to 70 datasets including nonobstacle images where considerable floor appearance changes. The quantitative segmentation accuracy is then compared with a conventional method, which we have also implemented. The accuracy of the distance estimation of the proposed method is also analyzed. 

The rest of the paper is organized as follows: we review the related works in Section 2. In Section 3, we provide an overview of the system. In Section 4, we introduce an improved IPM-based coarse obstacle detection method, followed by the obstacle segmentation and distance estimation method in Section 5. Section 6 presents the experimental results, and we conclude the paper in Section 7.

## 2. Related Work

In various environments, the floor can be locally considered to be a plane, and the detection of obstacles can be simplified to the problem of finding floor anomalies. Current vision-based algorithms can be classified into three categories: appearance-based methods, 3D reconstruction-based methods and homography-based methods.

For appearance-based methods, multiple visual clues from the environment are used for obstacle detection and free space detection. Lorigo *et al.* used color information in addition to edge information to differentiate between free space and obstacles [11]. Ulrich and Nourbakhsh proposed an obstacle segmentation algorithm based on a hue and intensity histogram [12]. These methods differentiate ground from obstacles by simply comparing the appearance of the image pixels to the appearance of the ground. Li and Birchfield [13] combined vertical edges, thresholding, and segmentation to approximate a wall–floor boundary and then classified the horizontal edges that lie on that boundary. Generally, purely appearance-based obstacle detection models fail when the floor texture changes or when the obstacle is extremely similar in appearance to the floor.

For 3D reconstruction based-methods, 3D information is extracted from point tracking for obstacle detection. Shen *et al.* [14] proposed a Lucas-Kanade-Tracker (LKT)-based time-to-contact (TTC) calculation for obstacle detection. Souhila *et al.* [15] proposed a Horn and Schunck dense optical flow-based TTC calculation for obstacle detection. The TTC-based method can be regarded as a simplified 3D reconstruction since these method can obtain depth information for each flow vector without doing full 3D reconstruction. Lalonde *et al.* [16] and Naito *et al.* [17] proposed full 3D reconstruction-based obstacle detection using an optical flow calculation. However, these works provide obstacle information only for tracked features. Furthermore, these methods require robust features for tracking in obstacle regions, and the accuracy of point correspondence affects obstacle detection performance.

For homography-based methods, most of the works track points near the floor, and obtain the cues for distinguishing between the floor and obstacle regions in an image from the floor homography error. Jin and Li [18] proposed a method for ground plane detection in which they used a dominant homography calculation between two images by classifying sparse feature points as floor or obstacle. Conrad *et al.* [19] used a SIFT feature tracking and homography-based modified expectation maximization algorithm to cluster pixels as belonging to one of two possible classes: ground or nonground. However, these works provide obstacle information only for tracked features, and require robust features for tracking in both ground and obstacle regions. 

More recently, segmentation methods have been combined with point tracking-based homography methods. These methods usually perform a purely appearance-based region segmentation first and combine homography information from point tracking, then the optimal label of image segments are determined. Specifically, Cui *et al.* [20] proposed LKT-based ground plane estimation based on homography combined with region-growing segmentation for obstacle labeling. Lin *et al.* [21] used k-means-based color segmentation combined with SURF feature tracking for ground labeling. Kumar *et al.* [22] proposed a LKT-based homography calculation for ground plane detection, which they then combined this with graph-based segmentation for floor extraction. More recently, Kumar *et al.* [23] proposed superpixel-based rough segmentation combined with homography information from LKT and vertical line detection for small obstacle detection. They formulated the problem as a Markov random field and used the graph cut algorithm [24] for optimal obstacle labeling. Although these methods combine image appearance and a homography-based geometry model, they still require robust points for tracking, especially for the obstacle region.

**Figure 1:**
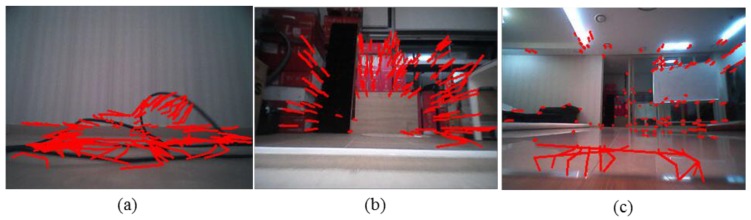
Tracking results for LKT [25] method when robot moves forward with no rotation. (**a**) Tangled wire. (**b**) Windowsill. (**c**) Floor with changing appearance.

As mentioned above, most of the prior works are based on point tracking. The point tracking-based methods suffer from the problem of false correspondence and require robust features to be tracked. However, for low-mounted cameras, point tracking near the floor becomes extremely difficult because the affine motion model, which is assumed by most of the algorithms, becomes invalid. Figure 1 shows the point tracking results of five sequential real indoor environment images when the camera moves forward with no rotational motion. The images are captured from a camera mounted at 6.3 cm above ground. For tracking, we use the pyramidal implementation of LKT scheme [25] employed by most previous works. A tangled wire case is shown in Figure 1a. Most of the tracked features are clearly false correspondences. A windowsill case is shown in Figure 1b. No tracked features appear in the windowsill region, which is the actual region of interest. Figure 1c shows a nonobstacle case where the floor appearance changes as a function of robot motion. The tracked features of the floor region clearly include false correspondences. These erroneous tracking results induce large homography errors and eventually lead to false positives. For robotic vacuums, false positives in obstacle detection mean poor cleaning performance.

**Figure 2:**
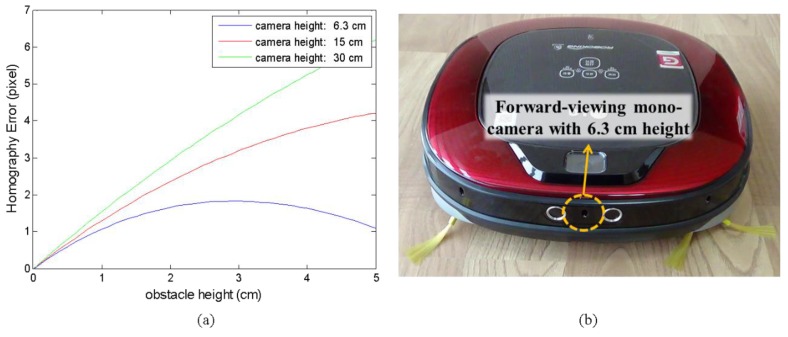
(**a**) Theoretical homography errors according to camera height when feature tracking is perfect. (**b**) Forward-viewing mono camera mounted at 6.3 cm above ground on a home service robot.

Furthermore, for low-mounted cameras, the homography errors from point tracking provide less-differentiable information. Figure 2a shows the theoretical homography errors as a function of obstacle height under the assumption of exact tracking and for several camera heights. The obstacle is assumed to be 0.5 m in front of the first camera, and the second camera moves 0.1 m forward with respect to the first camera. Evidently, it is much more difficult to distinguish obstacle from floor with a low camera. In this work, we use a camera mounted at 6.3 cm above ground, as shown in Figure 2b. 

For homography-based methods that do not use feature tracking, IPM-based methods can be used for obstacle detection. Originally, the IPM method was frequently used for eliminating the perspective effect of the original image in traffic stream detection or lane detection problems [26,27]. Few attempts were made to detect obstacles with monocular settings [28,29]. Neimann *et al.* [28] proposed an obstacle detection method for a vehicle application that estimates the ego motion and uses IPM. Jiang *et al.* [29] proposed a fast IPM algorithm for road surface recovery and obstacle detection for vehicle applications. Ma *et al.* [30] proposed a pedestrian detection algorithm that combines IPM detection with a vertical 1D profile to improve detection in the vertical direction and to overcome its limitation in a low-contrast environment. Since the IPM-based method does not use the point matching information, no risk of false correspondence exists. Furthermore, the IPM methods are based on the image warping from a floor homography model, not based on the homography errors. This provides more-differentiable information to distinguish the obstacle from the floor. In the method proposed herein, we combine the geometric cues from IPM and the appearance model for obstacle segmentation. For further performance improvement, a vertical plane model is adopted at the IPM stage.

## 3. System Overview

**Figure 3:**
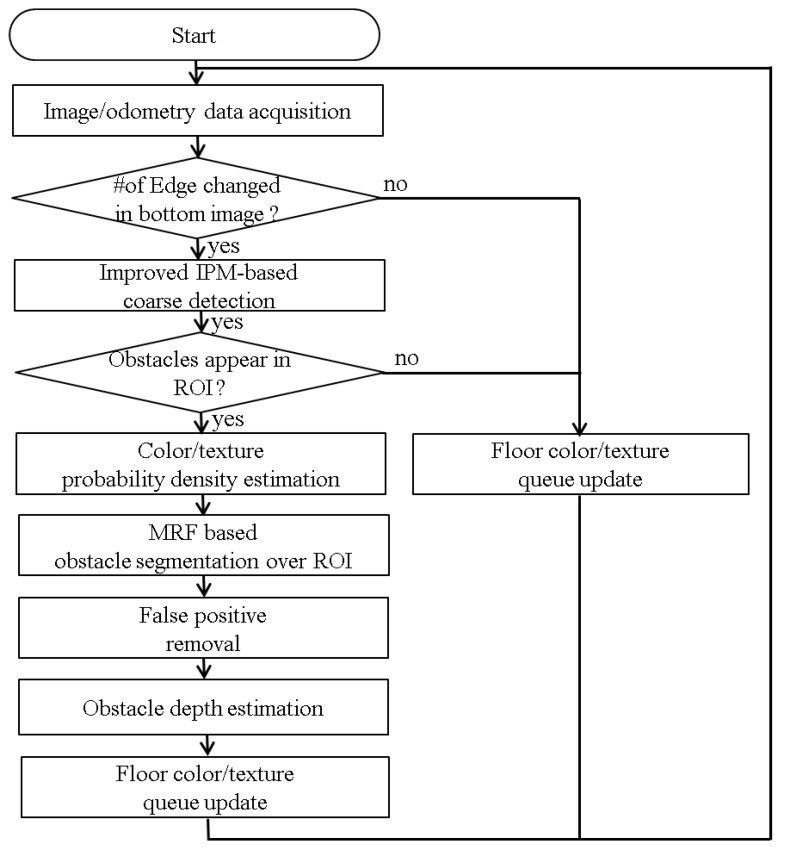
Flowchart of the overall approach.

In this work, it is assumed that the ground is relatively flat, and there are no overhanging obstacles in the environment. As sensory inputs, this study uses images captured from a forward-viewing mono camera and odometry from robot wheel encoders and gyroscope. The camera is 6.3 cm above the floor and slightly tilted by 7.2° to account for various consumer services such as monitoring or human-robot interactions. Figure 3 shows the overall flowchart of our approach.

In numerous cases, obstacles are placed more than 1 m in front of the robot. When the camera is low, the disparities in sequential images from the distant obstacle region reduce to nearly zero, which makes detecting far obstacles almost impossible. By skipping such images, unnecessary computation is avoided, which reduces the computation burden of the processor. For these reasons, we aim to detect near obstacles that appear in the bottom region of images. The algorithm passes over images by simply checking the variation of edge numbers in the bottom third of the image. This strategy is appropriate for robots that move at a speed less than 0.5 m/s, but it may not be applicable for fast-moving robots. When the robot moves toward a nearby obstacle, the number of edges in the lower image area usually increases because most obstacles contain edges. The simple change detection algorithm triggers the main obstacle detection algorithm to run for at least the three subsequent images. As the robot moves forward with no obstacle in the image, a change in the floor pattern will also trigger the main algorithm to run. In this case, the main algorithm distinguishes floor patterns from obstacles. 

After the main algorithm is triggered, improved IPM-based coarse obstacle detection is applied to the lower region of interest (ROI) image. If the lower ROI image is classified as a nonobstacle image, the obstacle segmentation step is skipped. Otherwise, the obstacle is segmented and the distance to the obstacle is estimated. The main novelties of this work are as follows. Firstly, a vertical plane model is adopted at the IPM stage for coarse obstacle detection. Secondly, the IPM-based method is combined with the color and texture appearance model for obstacle segmentation.

## 4. Improved IPM-Based Coarse Detection

### 4.1. Conventional IPM-Based Obstacle Detection

**Figure 4:**
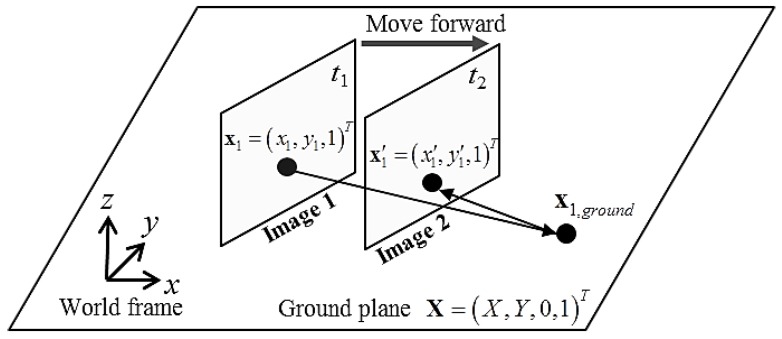
Illustration of IPM-based detection principle.

Figure 4 illustrates the geometrical properties of the camera image plane along with the corresponding transformed plane on the ground. A robot acquires images 1 and 2 at two different times *t*_1_ and *t*_2_, respectively, and can apply the relative transformation by using the odometry information between the two frames. Assume that pixel **x**_1_ in image 1 belongs to the floor. Then **x**_1_ can be projected to the floor as **x**_1,ground_, and then reprojected to image 2 as **x**’. This can be simplified as follows by using the floor homography matrix **H**:

(1) $${x^{\prime }}_{1}=H\cdot x_{1}$$

where **H** may be calculated by using the camera intrinsic matrix **K**, relative camera rotation **R** relative camera translation **t** floor normal **n** and the camera-floor distance *d* as follows:

(2) $$H=K\cdot (R-t\cdot n^{T}/d)\cdot K^{-1}$$

Likewise, assume that no obstacles exist under the horizon of image 1. Then all points of image 1 under the horizon can be warped, and the virtual image at time *t*_2_ can be generated. If this assumption is true, the virtual image at time *t*_2_ should be same as the real image 2. But if an obstacle exists under the horizon of image 1, the virtual image is no longer the same as the real image 2 of the obstacle area. In this way, obstacles are detected by simply subtracting the virtual image from real image 2. Because of its geometrical characteristics, the detection region of the IPM algorithm is limited to the region under the horizon, which means that, instead of evaluating the entire obstacle region, only the region under the horizon need be used for IPM detection. In this work, the obstacle detection region is set to be the lower vertical third and the middle horizontal three-quarters of the image. Any obstacle that appears in this region directly prevents the robot from moving forward. Hereinafter, we refer to this region as the ROI, and the algorithm applies IPM-based obstacle detection and obstacle segmentation inside this ROI. Note that, although trapezoidal regions are generally used to define the ROI, for simplicity we use a rectangular ROI in this research.

**Figure 5:**
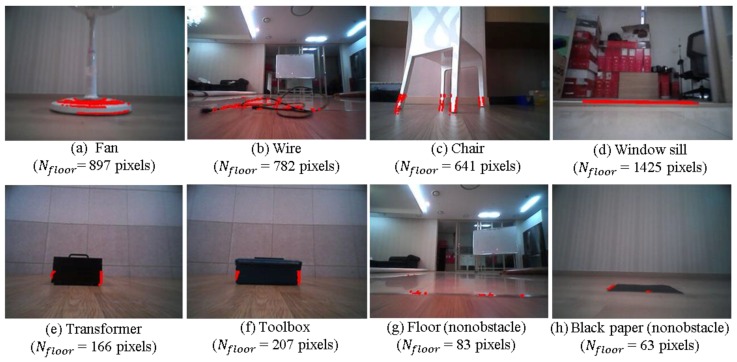
IPM-based obstacle detection results for various cases. Few obstacles are detected for the case of the transformer and toolbox in panels (**d**) and (**e**), whereas few obstacles are detected in the floor region in panels (**f**) and (**g**).

Generally, the IPM-based method can reliably detect most obstacles. Even though the camera is mounted at a low position in our case, the IPM-based method can detect any obstacle under the height of the camera when the obstacle is assumed to have a highly textured surface, and the floor homography model is accurate. This method completely avoids traditional feature extraction and matching or optical flow computation in obstacle detection. Although the IPM-based method is very efficient, it does suffer from two major drawbacks. First, few relatively large sized obstacles with homogeneous coloring or texture in their inner region are detected [31,32]. Second, errors in the homography model, unknown camera motion, and light reflection from the floor can introduce noise into the floor image. These drawbacks are well illustrated in Figure 5. The total number of pixels detected by the IPM methods as belonging to an obstacle is *N*_floor_. For the fan, wire, chair, and window sill illustrated in Figure 5a–d, the IPM provides reliable detection. However, little detection occurs for the boundary regions for the transformer and toolbox shown in Figure 5e,f. These obstacles are relatively large and have homogeneously colored inner regions. When the appearance of the floor changes as the robot moves, a few noisy detections occur in the floor region, as shown in Figure 5g,h.

With regard to this problem, a 1D vertical profile was proposed in [30] to distinguish true detections from noisy detections in IPM-based pedestrian detection. However, this method is based on the assumption that pedestrians have edges that are strongly vertically oriented compared with their background. However, this assumption is not valid in general, and no clear cue exists to distinguish correct detections from noisy detections at the pixel level. Instead, we classify the ROI image as obstacle image or nonobstacle image. Only after the ROI image is classified as an obstacle image, is the following obstacle segmentation conducted. However, as shown in Figure 5, simply classifying these two cases based on the number of detected pixels is not enough—further processing is needed.

### 4.2. Estimating Candidate-Obstacle Distance 

To distinguish a nonobstacle image from an obstacle image, we propose a vertical plane model-based image warping. To do this, the approximate distance between robot and candidate obstacle is required. As mentioned in the introduction, feature matching or optical flow based 3D reconstruction is not reliable for estimating obstacle distance. Instead, we propose a simple appearance-based method.

With monocular vision, a common approach to estimating distance is to assume that the ground is relatively flat and that no overhanging obstacles are present [12,33]. If these two assumptions are valid, the distance from the camera can be estimated for obstacle pixels in the lower region of the image because we know the camera height and tilting angle. In other words, if we figure out the lower region pixels of the obstacle, the approximate distance can be estimated based on the pinhole camera model. To further simplify the problem, we estimate the horizontal border line *l* in the ROI image that passes through the lower pixels of the obstacle. If we know the line *l* in the image, the ROI can be divided into two regions: an upper region Ω*_l,upper_* of the line *l* and a lower region Ω*_l,lower_* of the line *l*. The union of these two regions is the entire ROI image Ω*_ROI_* as follows:

(3) $$\Omega_{ROI}=\Omega_{l,upper}\cup \Omega_{l,lower}$$

**Figure 6:**
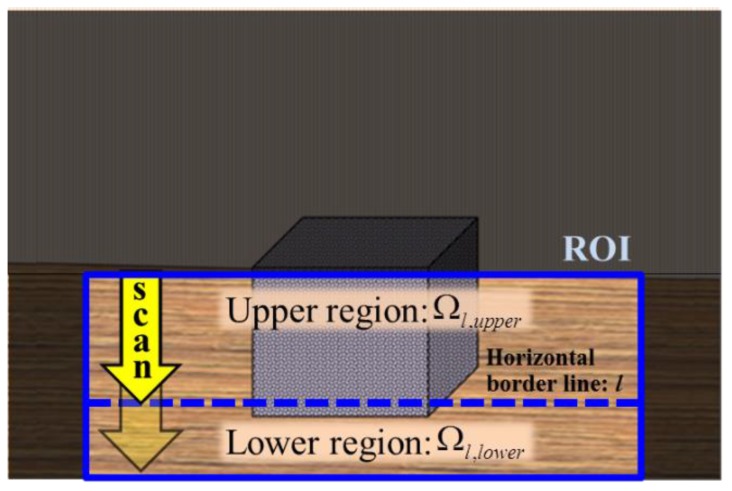
Formulation of problem for finding the horizontal border line that maximizes the difference in appearance between the upper and lower regions.

Next, we consider the appearance of the two regions. If the two regions have different color or texture distributions, they can be quantified as two probability density functions (pdfs). Among the possible lines *l* in the ROI region, the difference between two pdfs is maximized when the line *l* passes through the bottom pixels of the obstacle. Therefore, the distance estimation problem can be formulated as finding the horizontal border line *l* that maximizes the difference between pdfs of upper region and lower region. Figure 6 illustrates the problem formulation. 

**Figure 7:**
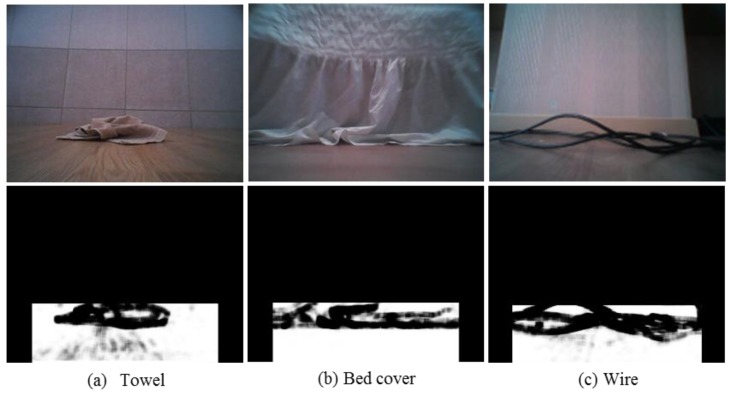
Metric tensor based texture feature extraction converted to 8-bit grayscale image in the ROI.

For the appearance model, we use the combined intensity and texture features. Of the various texture features, we use that proposed in [34] because it discriminates very well between foreground and background regions. The texture feature proposed in [34] is based on the geometry of textures using semilocal image information and tolls from differential geometry. We used a 6 × 6 square patch around pixel (*x,y*) for semilocal image information. The texture descriptor *T* is defined as:

(4) $$T=\exp\left(-\frac{\det(g_{xy})}{\sigma^{2}}\right)$$

where *g_xy_* is the metric tensor of the square patch and *σ*^2^ is a scaling parameter. In this work, *σ*^2^ is selected as one-thousandth of the maximum value of det(*g_xy_*) over the ROI image. The texture descriptor is especially useful when the obstacle texture differs from that of the floor. Figure 7 shows the extraction of texture description, which is converted to an 8-bit grayscale image for a towel, bed cover, and wire case. The results show that texture descriptors give highly differentiable information for dividing the obstacle region from the floor region.

Next, the joint probability density estimation of intensity and texture feature is conducted in the following way: Although relatively fast kernel density estimation methods exist such as the improved fast Gauss transform [35], a multidimensional nonparametric kernel density estimation in general requires high computing power. After careful consideration, we regard the intensity and texture feature as an independent variable in a naïve Bayesian manner. The joint probability density functions for a given region Ω*_l,upper_* and Ω*_l,lower_* can then be approximated in a simple form as:

(5) $$\{\begin{array}{c} f(I,T|\Omega_{l,upper})=f(I|T,\Omega_{l,upper})\cdot f(T|\Omega_{l,upper})≃f(I|\Omega_{l,upper})\cdot f(T|\Omega_{l,upper})\\ f(I,T|\Omega_{l,lower})=f(I|T,\Omega_{l,lower})\cdot f(T|\Omega_{l,lower})≃f(I|\Omega_{l,lower})\cdot f(T|\Omega_{l,lower}) \end{array}$$

where *I* and *T* are the intensity and texture features, respectively. Each intensity and texture pdf can be easily calculated by using a Gaussian kernel as follows:

(6) $$\{\begin{array}{c} f(I|\Omega_{l,upper})=\frac{1}{\left|\Omega_{l,upper}\right|}\int_{\Omega_{l,upper}}G(I-I(x))dx\\ f(T|\Omega_{l,upper})=\frac{1}{\left|\Omega_{l,upper}\right|}\int_{\Omega_{r,upper}}G(T-T(x))dx\\ f(I|\Omega_{l,lower})=\frac{1}{\left|\Omega_{l,lower}\right|}\int_{\Omega_{l,lower}}G(I-I(x))dx\\ f(T|\Omega_{l,lower})=\frac{1}{\left|\Omega_{l,lower}\right|}\int_{\Omega_{l,lower}}G(T-T(x))dx \end{array}$$

where |⋅| is the area of the given region, and *G*(∙) is the 1D Gaussian kernel with zero mean and variance *σ*^2^. To estimate the difference in appearance between the two regions, we use the Kullback-Leibler (KL) divergence. The KL divergence is frequently used to measure the distances between two pdfs [34,36]. The KL divergence between the pdfs of region Ω*_l,upper_* and Ω*_l,lower_* can be defined as:

(7) $$\begin{array}{l} KL(f(\Omega_{l,upper}),f(\Omega_{l,lower}))\\ =\int_{{\mathbb{R}}_{T}}\int_{{\mathbb{R}}_{I}}\left(f(I,T|\Omega_{l,upper}\right)\frac{f(I,T|\Omega_{l,upper})}{f(I,T|\Omega_{l,lower})}+(f(I,T|\Omega_{l,lower})\log\frac{f(I,T|\Omega_{l,lower})}{f(I,T|\Omega_{l,upper})})dI\cdot dT \end{array}$$

where ${\mathbb{R}}_{I}$ and ${\mathbb{R}}_{T}$ are the domain of the intensity and texture features. Next we find the horizon line that maximizes the KL divergence of two regions as follows:

(8) $$l^{*}=\underset{l}{\arg\max}\;KL(f(\Omega_{l,upper}),f(\Omega_{l,lower}))$$

By simply scanning the possible horizontal border lines within the ROI, we can find the $l^{*}$ that satisfies Equation (8). 

To reduce complexity, two strategies are adopted: The first strategy involves normalizing the intensity and texture feature domains ${\mathbb{R}}_{I}$ and ${\mathbb{R}}_{T}$ to integer values between 0 ≤ ${\mathbb{R}}_{I}$ < 64 and 0 ≤ ${\mathbb{R}}_{T}$ < 64, respectively. The second strategy involves using the downsampled image in finding l*. The scan is first done over a 1/16-sized downsampled image, and then the scan is done over the original sized image around the scanned line.

**Figure 8:**
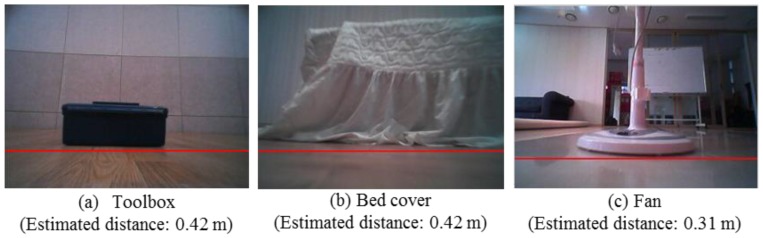
Estimated horizontal border lines that pass through the lower pixels of the obstacle.

This method takes only 5 ms to process on a common PC. Figure 8 shows typical examples of estimated horizontal border lines l*, which are marked as a red line. By assuming that the estimated border lines pass through the lower region of the obstacle, the estimated distances between the robot and the obstacle in Figure 8a–c are 0.42, 0.42, and 0.31 m, respectively

### 4.3. Decision for Obstacle Existence in the ROI Using Vertical Plane Model

**Figure 9:**
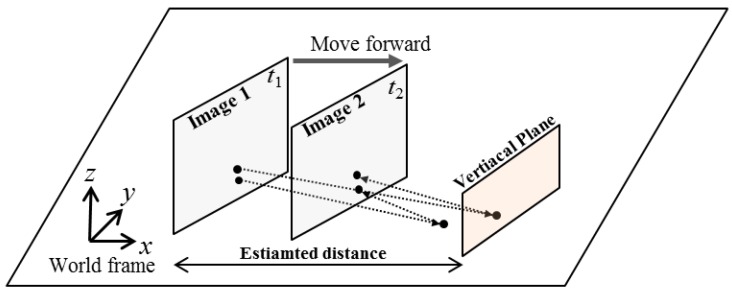
Illustration of virtual vertical-plane set up for IPM-based detection.

The previous section introduces the method for estimating the distance between the candidate obstacle and the robot. Next, the estimated distance is transformed with respect to the first frame, and a virtual vertical plane is set up. Figure 9 illustrates the geometrical properties of the camera image plane and the corresponding vertical plane. We assume that the obstacle can be modeled as a vertical plane. We calculate two homography matrixes: a floor homography matrix and the homography matrix of the obstacle’s vertical plane. By using these two homography models, the virtual image is calculated as done in the IPM method. The interpretation of this virtual image is that the world is composed of a floor and a vertical obstacle plane in front of the camera. 

In the subsequent step, the virtual image is subtracted from the real image 2 followed by thresholding and binarization. Next, the total number *N*_obstacle_ of detected pixels is counted. The total number of pixels detected by the IPM method is denoted *N*_floor_ because the IPM method models the ROI in image 1 as the floor. In contrast, the combined floor and virtual vertical planes model the ROI as a floor and a vertical plane shaped obstacle. The numbers *N*_obstacle_ and *N*_floor_ indicate the degree of inaccuracy of each model, respectively. Next, the ratio of *N*_floor_ to *N*_obstacle_ is used to classify the ROI image between the nonobstacle image and the obstacle image. If the ratio is large, it is reasonable to classify the ROI region as the obstacle image. Otherwise, the ROI region is classified as the nonobstacle image.

**Figure 10:**
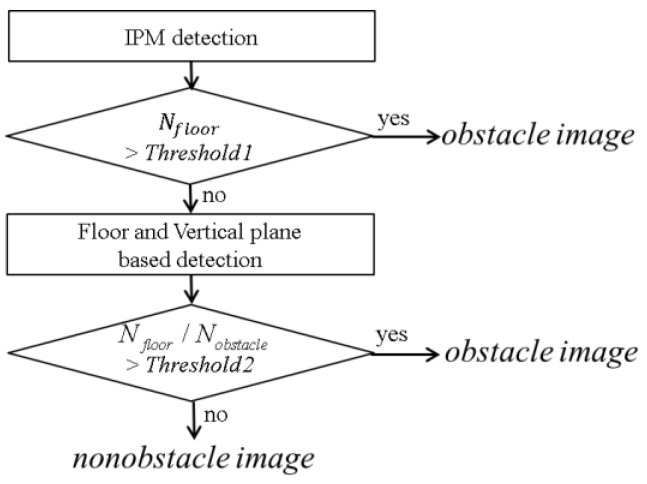
Decision rule for distinguishing between obstacle and nonobstacle images.

**Figure 11:**
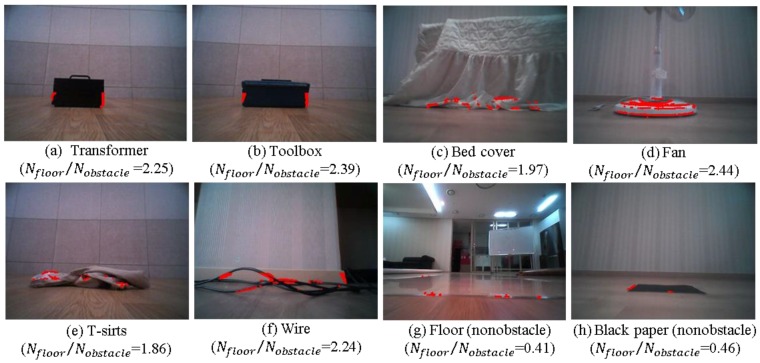
Examples of calculated ratio *N*_floor_/*N*_obstacle_ for various cases.

Figure 10 shows the proposed decision rule to distinguish between nonobstacle image and obstacle image. By setting appropriate thresholds, the two cases may be distinguished correctly. In our setting, *Threshold1* and *Threshold2* are determined experimentally to provide the best results as 300 and 1.5, respectively. These thresholds should be adjusted according to the height and tilting angle of the camera and image resolution. Figure 11 shows examples of calculated ratios for various cases. Interestingly, the fan, T-shirt, and wire seem unlikely to be modeled as vertical planes. However, these obstacles show a high ratio, as shown in Figure 11d–f. In addition, the cases where the appearance of the floor changes lead, as expected, to a low ratio, as shown in Figure 11g,h.

## 5. Obstacle Segmentation and Distance Estimation

From the improved IPM-based coarse obstacle detection process, the existence of obstacles at the ROI is known. The pixels detected by the IPM method provide a coarse cue about the obstacle. However, the exact obstacle boundary remains unknown. For fine obstacle segmentation, the algorithm does a Markov random field based obstacle segmentation by using the appearance of the obstacle and the floor. The proposed obstacle segmentation method is analogous to interactive segmentation, as used in [37]. Because we use appearance-based segmentation, false positives may occur, which are actually floor region areas labeled as obstacles. Using a morphological operation and IPM-detected data, false positives are removed. Next, the shortest distance between the robot and the obstacle is estimated by using the results of segmentation.

### 5.1. Appearance-Based Probability Density Estimation

For obstacle segmentation, the probability densities of floor and obstacle appearance are estimated. To model the floor appearance, the color histogram of the lower reference area of the image is frequently used [11,12,25]. Our algorithm for learning floor appearance is very similar to the adaptive method of Ulrich [12]. This method is very simple, reasonable, and practical in many robotic applications. We use the intensity and texture features as introduced in Section 4 to model each pixel’s appearance. We assume that the floor area over which the robot has already traveled is free of obstacles. A *candidate queue* and a *reference queue* are used for to update the adaptive floor appearance model. The reference queue is used to estimate the floor probability density in current obstacle segmentation. For our camera setting, the lower 15 rows of the image correspond to the floor 0.17 to 0.26 m in front of the robot. When no obstacle is detected in these 15 rows, the intensity, texture, and current odometry information are stored in the candidate queue. At each sample time, the current odometry information is compared with that from the candidate queue. Next, orientation variation is checked. Orientations that differ by more than 25° from the current orientation are eliminated from the candidate queue. Finally, the intensity, texture, and odometry information whose corresponding odometry positions differ by more than 0.26 m are moved from candidate queue into the reference queue. In the current reference queue, the old data whose position or orientation differ from the current position are removed because the floor appearance may change as the robot moves. The data whose corresponding odometry positions differ by more than 1 m or whose orientations differ by more than 45° are removed from the reference queue. 

The floor intensity and texture probability density from the reference queue is estimated only when the ROI of the current image is classified as an obstacle image by the improved IPM-based method. Otherwise, the candidate and reference queues are only updated. When the ROI of the current image is classified as an obstacle image at the beginning of robot operation or immediately after a rotation, the reference queue is not available. In this case, the intensity and texture information of the lower 15 rows of the image are immediately moved to the reference queue. A risk exists in such cases that the reference area for floor appearance model is not obstacle free. 

Estimating the obstacle appearance probability density is much easier than for the floor. We directly use the intensity and texture of the IPM-detected pixels. The estimate of the intensity or texture probability uses the same model as introduced in Section 4.2. The joint and conditional probability density can be approximated in a simple form as:

(9) $$\{\begin{array}{c} f(I,T|\Omega_{floor})≃f(I|\Omega_{floor})\cdot f(T|\Omega_{floor})\\ f(I,T|\Omega_{obstacle})≃f(I|\Omega_{obstacle})\cdot f(T|\Omega_{obstacle}) \end{array}$$

where the individual intensity and texture conditional probability can be calculated as follows:

(10) $$\{\begin{array}{c} f(I|\Omega_{floor})=\frac{1}{\left|\Omega_{floor}\right|}\int_{\Omega_{floor}}G(I-I(x))dx\\ f(T|\Omega_{floor})=\frac{1}{\left|\Omega_{floor}\right|}\int_{\Omega_{floor}}G(T-T(x))dx\\ f(I|\Omega_{obstacle})=\frac{1}{\left|\Omega_{obstacle}\right|}\int_{\Omega_{obstacle}}G(I-I(x))dx\\ f(T|\Omega_{obstacle})=\frac{1}{\left|\Omega_{obstacle}\right|}\int_{\Omega_{floor}}G(T-T(x))dx \end{array}$$

### 5.2. Markov Random Field Modeling

After estimating the probability density of the floor and obstacle appearance, the obstacle is segmented by using the Markov random field model. A Markov fandom field (MRF) is a probabilistic graphical model used to encode conditional dependencies between random variables. We pose our problem of segmenting the obstacle region in a MRF framework and define an energy function such that its minimum corresponds to the target segmented obstacle region in an image. In this framework, we represent each pixel of the image as a node in a Markov random field and associate a unary and pairwise cost of labeling these pixels. We then solve the problem in an energy minimization framework where the following energy function *E* is defined:

(11) $$E(x)=\sum_{i\in V}\psi_{i}(x_{i})+\sum_{i\in V,j\in N_{i}}\psi_{ij}(x_{i},x_{j})$$

where $\psi_{i}$ represents the unary term associated with pixel *i* and $\psi_{ij}$ represent the smoothness term defined over the neighborhood system N. Here **x** is the set of random variables corresponding to each pixel of the image in the ROI. Each of these random variables *x_i_* takes a label 0 or 1 based on whether it is a floor or an obstacle, respectively. From the probability model, the unary term of each pixel is:

(12) $$\psi_{i}(x_{i})=\{\begin{array}{c} -\ln f(I,T|\Omega_{floor})\;if\;x_{i}=0\\ -\ln f(I,T|\Omega_{obstacle})\;if\;x_{i}=0 \end{array}$$

$\psi_{i}$ may be interpreted as the closeness of each pixel to the floor and the obstacle based on the probability. The smoothness term is added only if the neighboring pixel has a different label as follows:

(13) $$\psi_{ij}(x_{i},x_{j})=\{\begin{array}{cc} \lambda \cdot \exp\left(\frac{-||I_{i}-I_{j}|{|}^{2}}{2\sigma^{2}}\right) & if\;x_{i}\neq x_{j}\\ 0 & if\;x_{i}=x_{j} \end{array}$$

Once the unitary and pairwise terms are defined, the problem of segmenting obstacles and floor depends on finding the global minima of the energy function defined in Equation (11):

(14) $$x*=\arg\min_{x\in L}E(x)$$

The global minimum of this energy function can be efficiently computed by using the graph cut method. We use the efficient, publicly available implementation of Kolmogorov *et al.* [24] to find the min cut of this graph. Since our graph cut-based obstacle segmentation is based on the color texture appearance model, a risk of obtaining false positives always exists. To remove false positives, we first morphologically open the system. Next, we group obstacle pixels by using connectivity. Finally, we label the entire group as floor when no IPM-detected pixel is inside the group. False positives can be removed efficiently with this method, leading to a reliable segmentation.

### 5.3. Estimating Obstacle Distance 

To avoid obstacles, the shortest distance between obstacle and robot must be estimated from the image. The coarse method of estimating obstacle distance, which was introduced in Section 4.2, is for distinguishing a nonobstacle image from an obstacle image. We require now a precise estimate of obstacle distance that uses the final obstacle segmentation results. As introduced in Section 4.2, a common approach to estimating distance involves using a monocular camera is to assume that the ground is relatively flat and that there no overhanging obstacles are present. If these two assumptions are valid, then the estimated distance to the bottom of a given obstacle is correct for all obstacles. However, the higher a given part of an obstacle is above the ground, the more overestimated is the distance. A simple approach to deal with this problem consists of grouping obstacle pixels and assigning the shortest distance to the entire group. An obstacle in any part of the ROI hinders the robot in its progression, so we are only interested in the shortest distance between the robot and the obstacle in the ROI. Therefore, the lowest part of the obstacle segmentation is important. To robustly find the lowest pixels from the noisy obstacle segmentation result, the algorithm uses a simple median filtering. We first store the height of the lowest obstacle pixels for each image column. Next, median filtering is done to find the lowest pixel in the image. Using the pinhole camera model, the shortest distance between robot and obstacle is easily calculated.

## 6. Experimental Results

This section presents the experimental results obtained by running the algorithm described above. The experiments use a typical robotic vacuum with a forward-viewing camera, wheel encoders, and a gyroscope. The robot platform is shown in Figure 2b. The images from a forward-viewing camera are collected at a resolution of 320 × 240 pixels at a 5 Hz data acquisition rate. Before conducting the experiment, the camera is calibrated by using the common checkerboard method [38], and the extracted intrinsic parameters are used in the visual data processing.

For the experiments, the following obstacles were selected: a wire, a fan, a speaker with a thin plate at the bottom, a chair with U-shaped legs, a four-legged chair, a bed cover, a window sill, a unicolor transformer, a unicolor toolbox, and a garment. We also tested four different nonobstacle cases where the floor changes appearance depending on the robot motion as follows: a floor material changes from marble to laminate and vice versa, a carpet changes from gray to pink, and a black A4-size paper lies on the floor. In this case the algorithm must be sufficiently robust to distinguish between the obstacle and any changes in floor appearance. 

The experiments are conducted in a typical home environment. The obstacles were laid at various places in the experimental environment. At the start of each test, the robot would be manually driven at 0.35 m/s toward an obstacle by using a remote controller. Each obstacle was tested five times and at various places. Each test dataset is composed of 15 to 20 images and odometry readings. Including the nonobstacle cases, the total number of datasets is 70 and the total number of images is 1079. 

**Figure 12:**
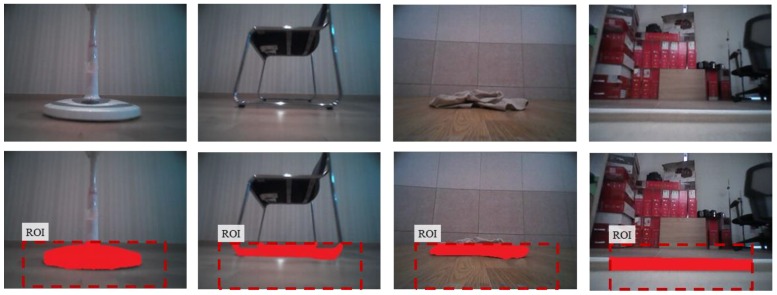
Examples of hand-labeled ground truth obstacle segmentation for quantitative analysis (upper row: original images, lower row: hand-labeled ground truth segmentation).

To quantitatively evaluate obstacle segmentation performance over an image domain, the obstacles were hand labeled for every image pixels to manually create a ground truth. Figure 12 shows examples taken from all the datasets of hand-labeled ground truth obstacle segmentation for quantitative analysis. Next, all pixels are classified into four classes: true positive (*TP*), true negative (*TN*), false positive (*FN*), and false negative (*FN*). We evaluated precision, false positive rate, and recall for all image data as follows:

(15) $$\{\begin{array}{c} Precision=\frac{TP}{TP+FP}\\ False\;Positive\;Rate=\frac{FP}{TP+TN+FP+FN}\\ Recall=\frac{TP}{TP+TN} \end{array}$$

Since the main algorithm is triggered based on edge information, the performance is evaluated only for images acquired after the main algorithm is triggered. The number of images to be evaluated was 412 from among 1079 total images. For nonobstacle datasets, all pixels in the lower ROI belong to the floor, and the obstacle pixels simply become false positives. 

To compare with conventional methods, we implemented a modified version of the model used in [23]. Originally, [23] used LKT-based optical flow but, as shown in the Figure 2, LKT is not suitable for low cameras. The original LKT-based method gives a disastrous result for obstacle detection in our experimental environment. Thus, we instead used the Gunnar–Farneback optical flow algorithm [39] for comparison, which provides better results in our environment. The best seed number for SLIC [40] at the ROI is determined experimentally to be 500.

**Figure 13:**
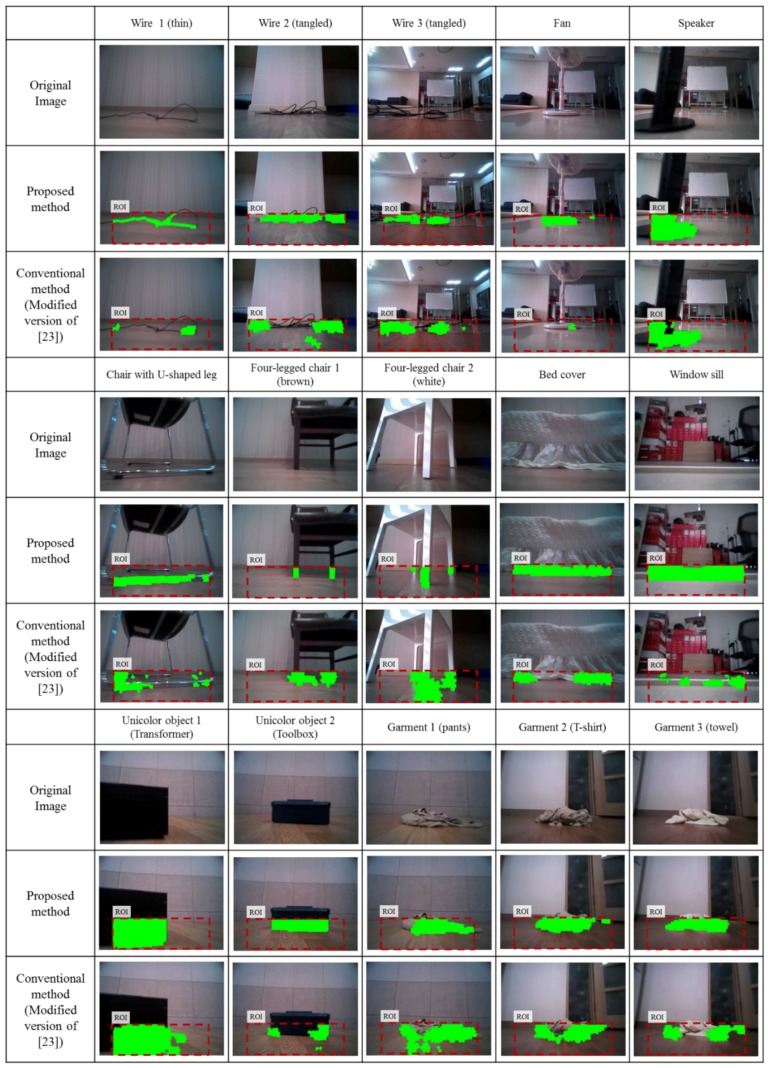
Obstacle labeling results from the proposed and conventional methods.

Figure 13 shows the results for obstacle labeling from among the 50 obstacle datasets. The first row shows the original image and the second and third rows show, respectively, the obstacle detection result obtained by using the proposed method and the conventional method (modified version of the method from [23]). Of these obstacles, the wire is the most hazardous for robot navigation. Wires 1, 2, and 3 in Figure 13 show three cases from the five wire datasets. Clearly, the proposed method detects the boundary of the obstacle much better than does the conventional method. In addition, the number of false positives with the proposed method is much less than with the conventional method. 

Table 1 shows the quantitative evaluations of obstacle segmentation and the error in distance estimation. For the proposed method, the precision, false positive rate, and recall are 81.4%, 5.9%, and 74.4%, respectively, whereas the results from the conventional method are 57.4%, 14.2%, and 37.6%, respectively. Even though the camera height is low above the floor, the image warping based IPM method can extract obstacle information reliably. Furthermore, combining the IPM-based method with the proposed appearance model leads to a better segmentation accuracy when compared to the conventional method. On the other hand, the conventional point tracking based method suffers from the false correspondence problem which leads to a decrease in segmentation accuracy. In addition, the calculated homography error from the point tracking provides less differentiable information which leads to a lower recall performance. 

By using the segmentation results, the algorithm to estimate distance gives the shortest distance from the robot to the obstacle based on the obstacle segmentation result. Next, the distance is transformed with respect to the initial robot position by using the current robot position. We then measure the distance estimation errors of the proposed method. Before each test, the shortest distance between the robot and the obstacle is manually measured for quantitative analysis. 

The total average error and the standard deviation of the proposed method are 1.6 and 5.8 cm, respectively, whereas those for a conventional method are 9.9 and 11.4 cm, respectively. As for the conventional method, the same distance estimation method introduced in Section 5.3 is used along with the corresponding segmentation results. Therefore, the difference in distance estimation error is attributed to the different performance level of obstacle segmentation.

**Figure 14:**
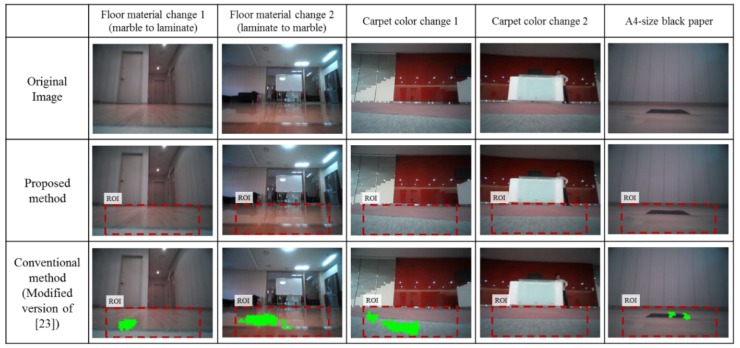
Obstacle segmentation results for nonobstacle images when the floor appearance changes.

Figure 14 shows the results of nonobstacle cases from the 20 datasets of four scenarios. The first row shows the original image and the second and third rows show the results obtained with the proposed method and with the conventional method (a modified version of the method used in [23]). The false positive rate of the result is shown in Table 2. The proposed method has a false positive rate of zero whereas the conventional method has a false positive rate of 17.6%. As mentioned in the introduction, the most serious problem of a high false positive rate for a robotic vacuum is that the robot regards the false positives as obstacles, which may result in poor cleaning. However, the proposed method efficiently differentiates between these cases by using the IPM method and proper thresholding.

Table 3 shows the computation time required to run the obstacle detection algorithm in each approach. The computation time is measured using an Intel Core i7-2600 running at 3.4 GHz. The proposed method and the conventional method take 87.9 ms and 136.0 ms, respectively. The simplicity of the proposed method, the improved IPM-based method and the appearance model, results in faster computation time compared to that of the conventional method.

## 7. Conclusions

This paper presented a monocular vision sensor-based obstacle detection algorithm for autonomous robots. Each individual image pixel in the lower region of interest is labeled as belonging either to an obstacle or to the floor based on the combination of geometric cues and the appearance model. Unlike state-of-the-art monocular vision based algorithms, which depend on feature tracking, the proposed algorithm uses the IPM method. This method is much more advantageous when the camera is low with respect to the floor, which makes feature tracking extremely difficult near the floor region. To further improve performance, a vertical plane model is adopted at the IPM stage. Using the IPM-based obstacle detection result and adaptive floor appearance learning, the algorithm conducts a Markov random field based obstacle segmentation. Next, the shortest distance between obstacle and robot is calculated. We evaluated the proposed method for 70 datasets including nonobstacle images where the floor appearance considerably changes. The quantitative analysis of segmentation accuracy and distance estimation accuracy were conducted and compared to the state-of-the-art method. The proposed method yields higher precision and better recall performance for the various obstacle datasets compared with the state-of-the-art method. It also has a false positive rate of zero for the nonobstacle datasets described in the paper. The proposed algorithm should be applicable to various kinds of autonomous robots for better navigation and path planning.

## Figures and Tables

**Table 1:** Segmentation accuracy and obstacle distance estimation error for obstacle datasets.

		Proposed Method	Conventional Method (Modified Version of [23])
Segmentation accuracy	Precision	81.4%	57.5%
	False positive rate	5.9%	14.2%
	Recall	74.4%	37.6%
Obstacle distance estimation error	Average error	1.6 cm	9.9 cm
	Standard deviation of error	5.8 cm	11.4 cm

**Table 2:** False-positive rate for nonobstacle datasets (change in floor appearance).

	Proposed Method	Conventional Method (Modified Version of [23])
False positive rate	0.0%	17.6%

**Table 3:** Computation time comparison.

	Proposed Method	Conventional Method (Modified Version of [23])
Computation time	87.9 ms (11.4 Hz)	136.0 ms (7.4 Hz)
